# Dosimetric comparison of pencil beam and Monte Carlo algorithms in conformal lung radiotherapy

**DOI:** 10.1002/acm2.12426

**Published:** 2018-08-05

**Authors:** Yelda Elcim, Bahar Dirican, Omer Yavas

**Affiliations:** ^1^ Department of Radiation Oncology Gulhane Training and Research Hospital Ankara Turkey; ^2^ Department of Engineering Physics Ankara University Ankara Turkey

**Keywords:** algorithm, Monte Carlo, pencil beam, radiotherapy, treatment planning systems

## Abstract

**Purpose:**

In this study, lung radiotherapy target volumes as well as critical organs such as the lungs, spinal cord, esophagus, and heart doses calculated using pencil beam (PB) and Monte Carlo (MC) algorithm‐based treatment planning systems (TPSs) were compared. The main aim was the evaluation of calculated dose differences between the PB and MC algorithms in a highly heterogeneous medium.

**Methods:**

A total of 6 MV photon energy conformal treatment plans were created for a RANDO lung phantom using one PB algorithm‐based Precise Plan Release 2.16 TPS and one MC algorithm‐based Monaco TPS. Thermoluminescence dosimeters (TLDs) were placed into appropriate slices within the RANDO phantom and then irradiated with an Elekta‐Synergy^®^ Linear Accelerator for dose verification. Doses were calculated for the V5, V10, V20, and mean lung doses (MLDs) in bilateral lungs and D50, D98, D2, and mean doses in the target volume (planning target volume, PTV).

**Results:**

The minimum, maximum, and mean doses of the target volumes and critical organs in two treatment plans were compared using dose volume histograms (DVHs). The mean dose difference between the PB and MC algorithms for the PTV was 0.3%, whereas the differences in V5, V10, V20, and MLD were 12.5%, 15.8%, 14.4%, and 9.1%, respectively. The differences in PTV coverage between the two algorithms were 0.9%, 2.7% and 0.7% for D50, D98 and D2, respectively.

**Conclusions:**

A comparison of the dose data acquired in this study reveals that the MC algorithm calculations are closer to the 60 Gy prescribed dose for PTV, while the difference between the PB and MC algorithms was found to be non‐significant. Because of the major difference arising from the dose calculation techniques by TPS that was observed in the MLD with significant medium heterogeneity, we recommend the use of the MC algorithm in such heterogeneous sites.

## 1 INTRODUCTION

The primary goal of radiotherapy was to achieve maximal tumor control with the optimal dose distribution while minimizing normal tissue exposure to avoid treatment‐related complications. Many different medical linear accelerators and treatment planning systems (TPSs) along with different algorithms provided by several vendors are available in the modern radiotherapy era to help reach this goal.

Pencil beam (PB) and Monte Carlo (MC) are among the avaliable algorithms. Regarding photon beams, one of the most used calculation algorithms implemented in 3DCRT (three‐dimensional conformal radiotherapy) is PB, and the most preferred algorithm of intensity‐modulated radiotherapy (IMRT) is MC.

The algorithms used in radiotherapy treatment planning are categorized as correction‐based, model‐based, and MC. These algorithms have advantages or disadvantages in the calculation of the absorbed dose, particularly in cases of a transition from one medium to another, a tissue‐air interface, the skin entrance or small‐irregular treatment fields. Any of these algorithms may be used in 3DCRT planning, although they may have different speeds and accuracies. To meet the International Commission on Radiation Units (ICRU) criteria, the dose calculation accuracy must be within 2%–3%.^1^

PB is a correction‐based algorithm in which dose calculation is performed on an infinitely narrow pencil beam dose distribution. Dose kernels are acquired within a homogeneous medium such as water to calculate the absorbed dose. The PB algorithm takes into account the inhomogeneity correction for the longitudinal direction in the central beam axis but ignores lateral scatter. This condition may cause dose calculation inaccuracies for heterogeneous treatment sites such as the lung or chest wall. A patient's body contains different densities, requiring a correction factor for each beam causing beam attenuation.^2^ The PB algorithm is very fast due to its use of a one‐dimensional density correction, which does not accurately model the distribution of secondary electrons in heterogeneous media,^3^, ^4^ while its limitations in a heterogeneous medium are well known. The PB algorithm considers collimated photon beams incident on the patient as clusters of many small, narrow “pencil beams” each of which has central axis ray deposits with certain doses. The pattern of such dose deposits changes with the intensity and the beam spectrum incident on the patient.^5^

MC is an algorithm in which mathematical phantoms are used to handle practical difficulties. Organ dose calculations are executed by performing a mathematical simulation of x ray interactions. In an MC algorithm, the distance traversed in a phantom or patient body by each photon of a certain x ray spectrum is monitored, and the energy released to the medium is detected using the interaction probabilities through its track. MC is the most accurate treatment planning method, but it is often not practical because it requires undesired long computational times.^6^ The linear accelerator head, including its components, is simulated with the MC algorithm since model‐based dose calculations require a great deal of attention to the details of the photon transport in the linear accelerator head. The fluence and energy distribution of photons emerging from the accelerator can be obtained with MC.^7^ The MC algorithm, which is considered the gold standard for dose calculation,^8^, ^9^ is the only dose calculation algorithm that can properly account for the lack of electronic equilibrium and the secondary buildup. However, the long calculation time required for MC calculations with present‐day computers makes it impractical for application in a clinical setting, and routine patient dose calculations are performed with model‐based algorithms.^10^ The MC technique explicitly tracks electron transport and is generally considered the gold standard in determining the electron disequilibrium dose distribution.^11^, ^12^

The calculation of dose distributions in inhomogeneities is a challenge; inhomogeneity corrections are needed in the air cavities, lung, bone and high‐density environments, and these corrections should be implemented in clinical applications.^8^ This step is even more important in high‐density inhomogeneous media such as the lung and head and neck region, where air cavities are present. Low‐density lung tissue can result in reduced photon attenuation and an increased range of secondary electrons, which can cause the algorithm to fail to accurately calculate the absorbed dose.^13^

The purpose of this study was to address the existing problems related to dose calculations in a heterogenous medium to indicate the discrepancy between DVHs acquired by PB and MC algorithms using TLD measurement verification.

## 2 METHODS

In this study, 93 computed tomography images of the Alderson RANDO phantom (RSD Radiology Support Devices, Long Beach, CA,USA) were acquired via a General Electric (GE) Light Speed Radiotherapy device with a thoracic arms‐up protocol, SFOV (scan field of view) of 50 cm, standard resolution, 888 × 1192 matrix size and 3.8 mm slice thickness. Target volumes and critical organs were both delineated with an Advance SimMD contouring Workstation. Two different treatment plans were created using 6 MV photon beams with PB and MC algorithms. For the PB algorithm, we used the Elekta Synergy^®^Linear Accelerator including 80 leaves of MLC (multileaf collimator) of 1 cm thicknesses at 100 cm SSD with Precise Plan Release 2.16 TPS, for the MC algorithm, we used the Elekta Synergy^®^ Linear Accelerator including 160 leaves of MLC of 0.5 cm thicknesses at 100 cm SSD with Monaco Version 5.10 TPS.

**Figure 1:**
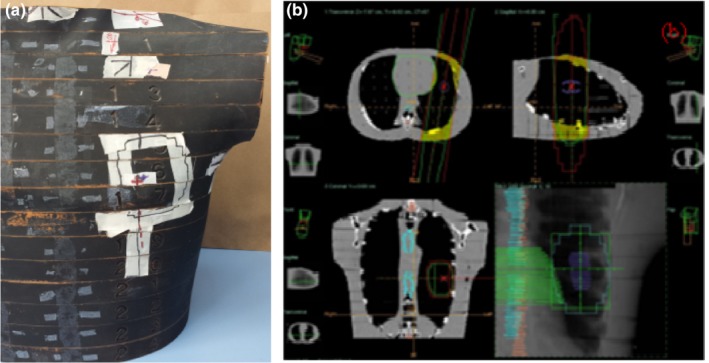
Image (a) shows a physical view of the lung treatment field, and (b) shows treatment planning views of transverse, sagittal, coronal slices with a DRR image of a RANDO phantom.

The left side of Fig. 1 shows the RANDO phantom lung treatment site, and the right side shows a treatment plan created by Precise Plan. Gross target volume (GTV), clinical target volume (CTV), and planning target volume (PTV), along with critical organs such as the lungs, spinal cord, heart and esophagus, were contoured. TLDs were numbered and placed at predefined locations within the treatment site and field edges in the RANDO phantom, as shown in Table 1.

**Table 1:** PB and MC algorithm dosimetry and TLD verification

RANDO phantom slice No.	TLD No.	TLD location	PB Dose (cGy)	TLD (cGy)	% difference	MC Dose (cGy)	TLD (cGy)	% difference
15	7	7 mm from the skin	23.14	92.37	−75	93.3	122.9	−24.1
16	3	15 mm from the skin, lung edge	206.09	190	8.5	204	193.3	5.5
16	4	5 cm from the skin, lung, field edge	34.07	32.74	4.1	64.6	61.98	4.2
16	5	9 cm from the skin, heart	7.62	7.24	5.3	7.1	6.75	5.2
16	6	7 cm from the skin, lung, center	199.38	196.1	1.7	193	190.8	1.2
17	2	5 cm from the skin, lung	190.18	193.7	−1.8	190.4	192.3	1.0
18	1	5.5 cm from the skin, heart; out of field 1 cm	10.66	12.56	−15.1	24.9	29.24	14.9

Treatment plans were created using identical parameters (1 cm margin for PTV, two‐opposing fields, the same fields and angles of the beams, the same normalization procedure and the same isocenter depth as that for PTV, 2 Gy/fraction and a total prescribed dose of 60 Gy) to acquire the dose distributions. Since the delineation of target volumes and critical organs may affect the DVH values, we used the same contours of target volumes and critical structures for a robust comparison between the algorithms. We comparatively assessed the maximum dose, minimum dose, mean dose, and mean lung dose (MLD); V5, V10, and V20 (volumes that receive 5, 10, and 20 Gy, respectively); and D50, D98, and D2 (dose values that 50%, 98%, and 2% of the organ volumes of interest receive, respectively) of the target volumes and critical organs extracted from the DVHs.

The dosimetric verification of the PB and MC algorithms for the treatment site was performed with RANDO Phantom and TLD‐100 chips. The TLD‐100 chips used for dose measurements were heated to 400°C for 1 h and then irradiated to 50 cGy at 100 cm SSD and 5 cm phantom depth at a 10 × 10 cm^2^ field size for calibration and labeling. The WinRems program and Harshaw 3500 TLD reader were used in the acquisition of dose values. TLD‐100 chips with ≤ 1% standard deviation were chosen for this study. The TLD measurements were repeated three times, and the mean dose results are given in Table 1.

After isocenter verification at the AP and LAT positions using Iview image guidance, irradiation was performed on the phantom according to the appropriate plan shown in Fig. 1. Figure 1(a) shows a physical view of the lung treatment field, and Fig. 1(b) shows treatment planning views of transverse, sagittal, and coronal slices with a DRR image of the RANDO phantom.

**Figure 2:**
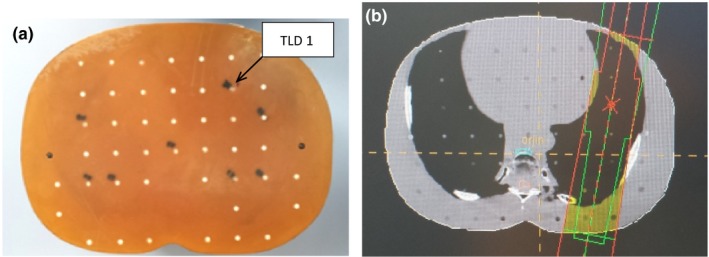
(a) RANDO Phantom slice No. 18 of TLD No. 1; (b) Treatment planning images of (a).

**Figure 3:**
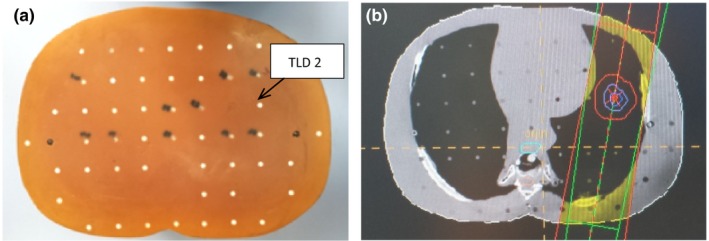
(a) RANDO Phantom slice No. 17 of TLD No. 2; (b) Treatment planning image of (a).

**Figure 4:**
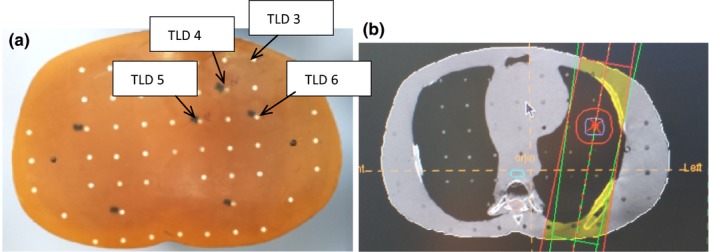
(a) RANDO Phantom slice No. 16 of TLD Nos. 3–6; (b) Treatment planning image of (a).

**Figure 5:**
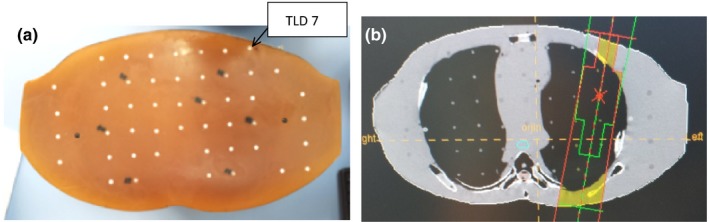
(a) RANDO Phantom slice No. 15 of TLD No. 7; (b) Treatment planning image of (a).

These procedures were repeated for both the PB and MC algorithms. Figures 2(a) and 2(b), 3(a) and 3(b), 4(a) and 4(b) and 5(a) and 5(b) show specific TLDs assigned to specific pulmonary phantom slices and relevant treatment planning images.

## 3 RESULTS

**Figure 6:**
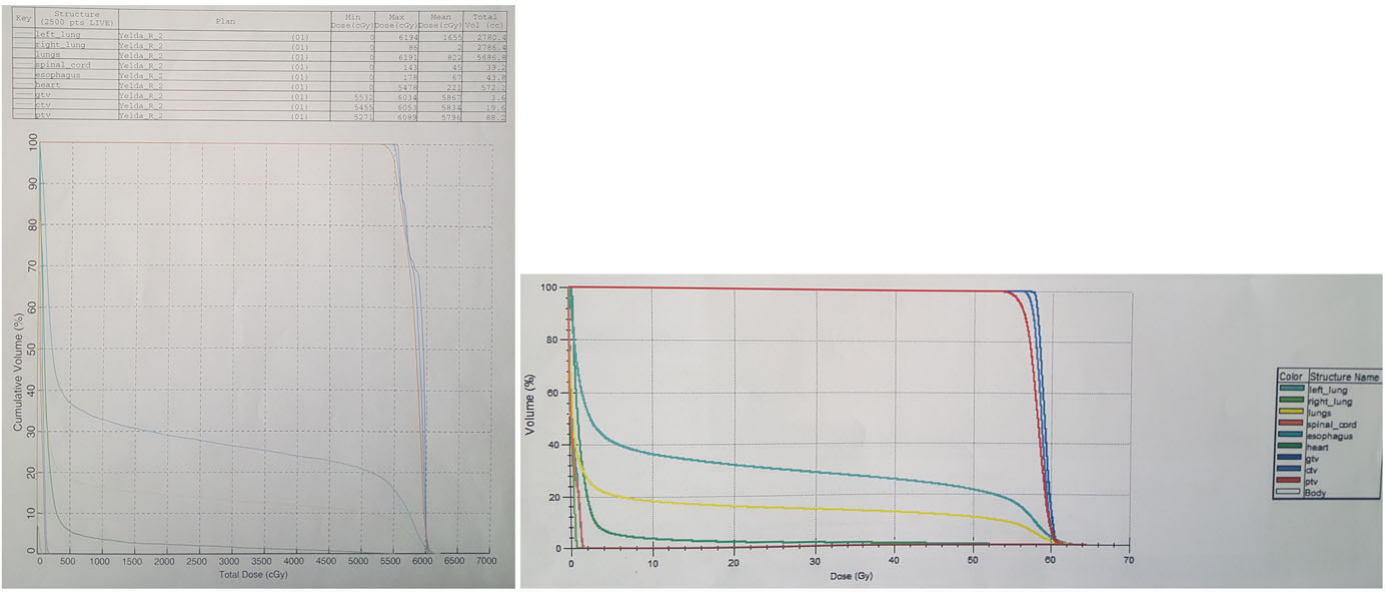
DVHs derived from the PB algorithm with Precise Plan (on the left) and the MC algorithm with a Monaco treatment plan (on the right).

The comparative assessment of point doses calculated by TPSs using the PB and MC algorithms are shown in Table 1. The doses measured by TLD no. 7 reveal that the calculated skin entrance dose difference at 7 mm depth was 21.2% and 24.1% using the PB and MC algorithms, respectively. The doses measured by TLD no. 3 indicate that the calculated dose difference at lungs edge within 1.5 cm depth was 8.5% and 5.5% using PB and MC algorithms, respectively. The doses measured by TLD no. 4 demonstrate that the calculated dose difference in the lungs at 5 cm depth and at the field edges were approximately 4% for both algorithms. The doses measured by TLD no. 5 reveal that the calculated dose difference in the lungs at 9 cm depth and at the heart was approximately 5% for both algorithms. The doses measured by TLD no. 6 show that the calculated dose difference in the lung field center at 7 cm depth was 1%–2% for both algorithms. The doses measured by TLD no. 2 indicate that the calculated dose difference in the lung at 5 cm depth was 1%–2% for both algorithms. The doses measured by TLD no. 1 reveal that the calculated dose difference in the heart at 5.5 cm depth and 1 cm outside the field was approximately 15% for both algorithms. DVHs derived from the PB algorithm with Precise Plan (on the left) and the MC algorithm with Monaco treatment planning (on the right) are shown in Fig. 6. Table 2 shows the minimum dose, mean dose, and maximum dose parameters for GTV, CTV, PTV, heart, esophagus, spinal cord, lungs, left lung, and right lung.

**Table 2:** DVH evaluations for PB and MC algorithms in planning lung 3DCRT

Structures	Minimum dose (cGy)		Maximum dose (cGy)		Mean dose (cGy)	
	PB	MC	PB	MC	PB	MC
GTV	5532	5698	6034	6090	5867	5910
CTV	5455	5599.3	6053	6107	5834	5867
PTV	5271	5305	6089	6206.2	5796	5815
Heart	0	37.6	5478	5894.3	221	234.3
Esophagus	0	0	178	168.9	67	552
Spinal cord	0	0	143	159.8	45	470
Lungs	0	0	6191	6438.3	822	904
Right lung	0	0	86	970	2	258
Left lung	0	8.1	6194	6438.3	1655	1786

**Figure 7:**
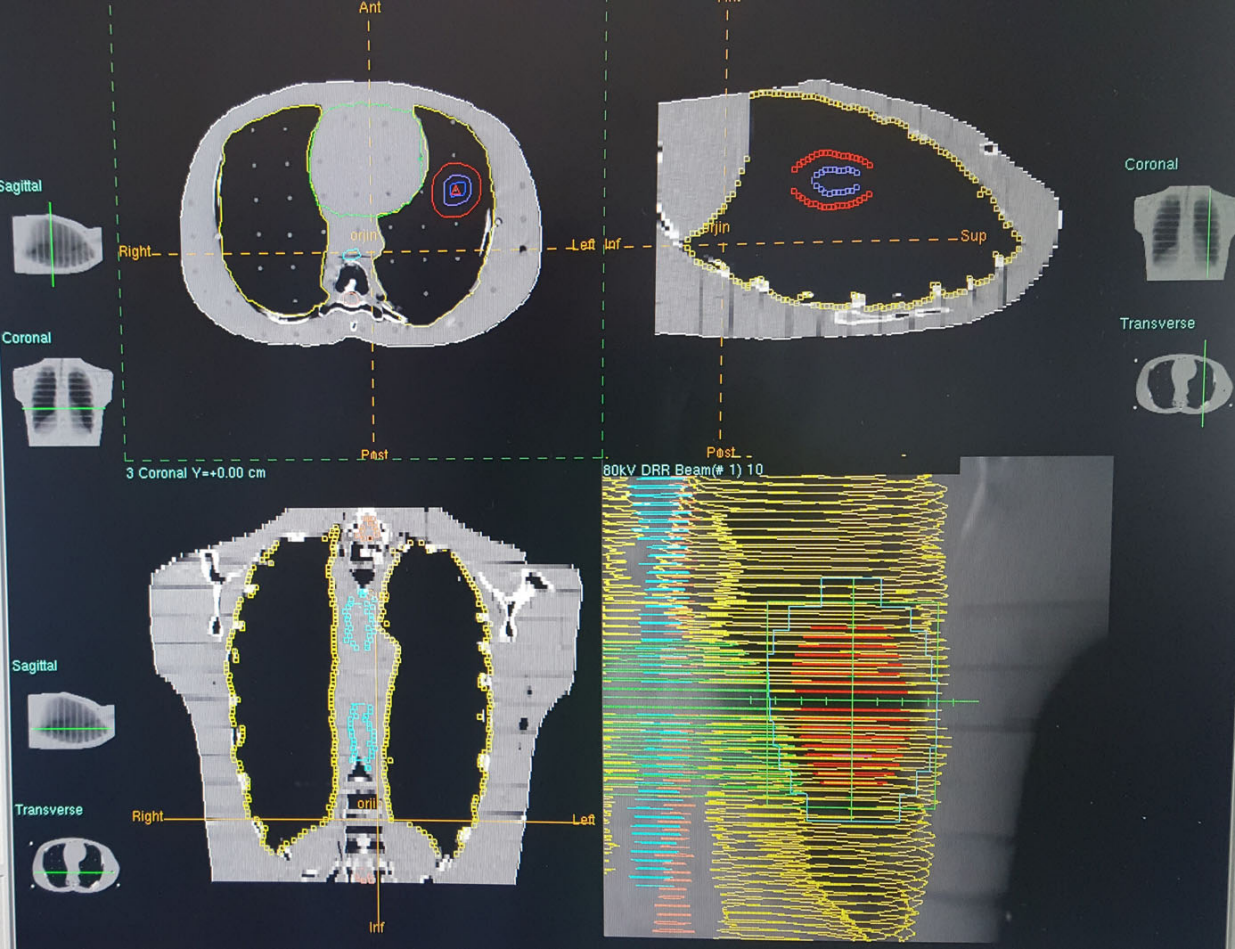
Transverse, sagittal, coronal dose distributions, and DRR views of the PB algorithm Precise Plan Release 2.16.

**Figure 8:**
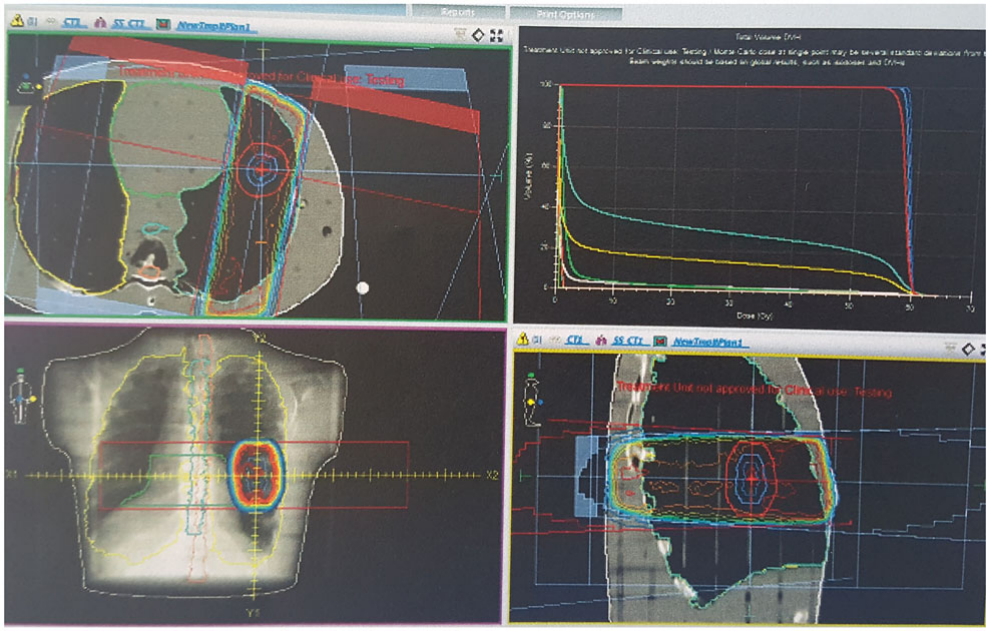
Transverse, sagittal, coronal dose distributions, and DVH views of the MC algorithm Monaco Version 5.10.

The dose distribution of the treatment plans of our study group revealed different isodose color washes; for example, the 100% isodose curve of PB does not go beyond the skin entrance, whereas the MC 100% isodose curve extends to the target volume, visually passing the skin entrance. The shapes and color washes of the other isodose curves washes similarly differ, as shown in Figs. 7 and 8. The treatment plans were evaluated using the DVH and the dose distributions together. Table 3 shows the D50, D98 and D2 dose parameters for the PTV and MLD and V5, V10, and V20 parameters for the lungs with comparative assessments of the 3DCRT treatment plans using the PB and MC algorithms.

**Table 3:** Dose analyses with PB and MC algorithm 3DCRT treatment plans

Lungs		PB	MC
	V20	14%	16%
	V10	16%	19%
	V5	18%	21%
	MLD	822 cGy	904 cGy
PTV	D50	58.5 Gy	58 Gy
	D98	54.5 Gy	56 Gy
	D2	60.6 Gy	61 Gy

## 4 DISCUSSION

We compared the PB and MC algorithms with different leaf thicknesses because we used two different linear accelerators and therefore provided a 1 cm margin to avoid the thickness and shape effects of the leaves on the dose distribution and color washings. We also used the AAPM TG‐142 Report of the quality assurance of medical accelerators^15^ to mechanically and dosimetrically compare two different linear accelerators.

We used TLD‐100 dosimeters in the dose verifications since the effective atomic number of TLD‐100 dosimeters is considered equivalent to that of soft tissue, making this a suitable clinical dose measurement tool. Differences between the TLD and PB and between the TLD and MC are consistent, as shown in Table 1. The results show the following:

- TLD no. 7: the skin entrance dose measured at 7 mm depth indicates that TLD calculations were higher than both the PB and MC algorithms (74.9% and 24.1%, respectivly). This finding indicates that both algorithms undercalculated the skin dose. The MC algorithm provides a more accurate calculation of the skin dose than does the PB algorithm.
- TLD no. 3: the dose measured at 1.5 cm depth at the lung edge heterogeneity transition point reveals that the MC algorithm provides a more accurate calculation of the dose than does the PB algorithm with a 5.5% difference.
- TLD no. 4: the dose measured at 5 cm depth at the lung field edge shows a difference between the PB and MC algorithms, possibly due to the difference in the MLC leaf width.
- TLD no. 5: the dose measured at 9 cm depth at the heart indicates that the dose calculations are consistent in both algorithms (7.62 and 7.1 cGy).
- TLD no. 6: the dose measured at 7 cm depth in the lung and field center reveals that the MC‐TLD dose difference is less than that of the PB‐TLD; therefore, the MC algorithm calculates the more accurate dose (1.7%–1.2%).
- TLD no. 2: the dose measured at 5 cm depth at the lung indicates that the algorithms calculate identical doses (190.18 and 190.4 cGy).
- TLD no. 1: the dose measured at 5.5 cm depth at the heart and 1 cm outside the field reveals that the MC algorithm includes less protection with a higher calculated dose than dose the PB algorithm as a result of beam penumbra and leaf width differences.

DVH analysis is one of the most important processes for evaluating the entire radiotherapy plan. Because the DVH values for both the critical organs and target volumes are calculated by the TPS, the dose calculation accuracy within a reasonable amount of time is a vital component of the algorithm. A notable point in the assessment of critical organ doses is that the maximum dose is very important for sequential organs, and the mean dose is more important for parallel organs.

Table 2 shows that the dose difference between the PB and MC algorithms is <1% for the target volume mean doses, revealing that the 3DCRT of the lung with both algorithms provides comparable calculations (0.7%, 0.6%, and 0.3% for the GTV, CTV, and PTV, respectively), which is not the case for critical organs. An evaluation of the critical mean organ doses reveals one interesting fact: the differences between the two algorithms are minor (7.3% and 5.7% for the left lung and heart, respectively) depending on the critical organ's proximity to the treatment site. However, if the location of the critical organ is distant from the treatment site, the difference increases dramatically (87.9%, 90.4%, 99.2% for the esophagus, spinal cord and right lung, respectively). The reason for this difference is that when the location of the critical organ in question is not directly within the beam axis, the PB algorithm calculates an underdose due to it is lack of a lateral scatter calculation. This undercalculation may mislead the physicist and physician, causing them to have less concern than they should in terms of clinical toxicities during the treatment planning phase as well as future considerations such as re‐irradiation.

Our study reveals that calculations using the PB and MC algorithms are reliable and consistent with each other in terms of the mean dose for the target volume; additionally, the results of both algorithms were generally in good agreement with the measurements. Minimum dose values are important for assessing target volume coverage; that is, higher minimum doses indicate improved target volume coverage. As shown in Table 2, the difference between the MC and PB algorithms with regard to the GTV, CTV, and PTV minimum doses were 2.9%, 2.6%, and 0.6%, respectively. The PTV minimum dose was better with the MC algorithm, but the difference was not significant.

Inhomogeneous transition areas, especially the skin entrance, provide another challenge for algorithms to overcome. If minimum doses are calculated as 0 cGy, indicating no dose at all, then the algorithm may mislead the user by undercalculating the target mean doses. The PB algorithm failed to calculate all the minimum critical organ doses and gave a calculated dose result of zero (0) cGy. However, the MC algorithm calculated minimum left lung and heart doses of 8.1 and 37.6 cGy, respectivly, while other critical organ minimum doses were zero (0) cGy. The minimum doses of zero (0) cGy in the critical organs calculated by the algorithms affect the mean doses to those organs.

Table 2 shows that the MC algorithm calculates a higher target volume maximum dose; however, the difference between the algorithms was <1% for the GTV and CTV and <2% for the PTV, which may be considered negligible and compatible; therefore, this difference clinically irrelevant because the maximum dose values are within the target volumes.

The minimum, maximum, and mean doses of the target volumes and critical organs in two treatment plans created by one TPS using MC and the other using PB were compared using the DVHs. The conformal lung radiotherapy mean dose differences between the PB and MC algorithms for the target volumes were 0.7%, 0.6%, and 0.3% in GTV, CTV, and PTV respectively; for the bilateral lung doses, these values were 12.5%, 15.8%, 14.4%, and 9.1% in V5, V10, V20, and MLD, respectively. The PTV coverage differences between the 2 algorithms were 0.9%, 2.7%, and 0.7% in D50, D98, and D2, respectively.

The current algorithms used in commercial TPS have advantages over others based on certain aspects such as calculation time versus calculation accuracy. In recent years, several studies have been conducted to allow this comparision.^16^ The thoracic region anatomy includes structures with different densities such as the chest wall, heart, lung, and bone. This variety of densities creates inhomogeneous medium transitions, which present a challenge for TPS algorithms to accurately predict the delivered dose. The difference between the delivered and predicted doses can be up to 20% in both the target volumes and critical organs^17^, ^18^, ^19^ that may require the reevaluation of treatment planning as well as the clinical outcomes when treatment is complete.

In the study by Kry and et al.,^21^ the PB algorithms were compared with the CS, AAA and MC algorithms at the treatment field isocenter and the PB dose calculation was found to be overestimated by 5% compared to the MC. The MC algorithm showed a deviation of 6% in the measured values and gave the most accurate value. Similar to the previous study, we found a 3% the PB overestimation between the PB and MC algorithms at the treatment field isocenter. In the study by Neisen et al.,^22^ six different algorithms were evaluated in four different TPSs in the lung, and no difference was found between the algorithms for the lung V20 and MLD doses.

The absorbed dose in heterogeneous media is dependent on the selected treatment beam energy, with a significant increase expected above 10 MV. The study by Imad et al.,^23^ with a setting similar to ours, revealed a 40% calculated dose difference for V95 6 MV irradiation between the PB and MC algorithms, with an overestimated PB for lung tumors. We studied V5, V10, and V20 for the lungs from individual PB and MC treatment plan DVHs. Our study showed significant calculated total lung dose differences in favor of the MC between the PB and MC algorithms, prominently in the MLD as well as V20, V10, and V5, with values of 10%, 2%, 3%, and 3%, respectively. The PB algorithm undercalculated the total lung dose, which may alter the treatment decision‐making progress.

Using the PB and MC algorithms for the 3DCRT of the lung, we found a significant calculated dose difference in the MLD of 9% due to inhomogeneities. Dose calculations with the PB and MC algorithms typically do not show wide diversities except for heterogeneous areas such as the lungs. In the 3DCRT, both the PB and MC algorithms may be used if the treatment field does not include heterogeneities. Our results are consistent with the study by Yangun et al.^24^ The magnitude of the difference may be higher in the case of small treatment volumes and negligible in the case of large GTVs. In our study, the treatment volume was not small; however, inhomogeneities due to air‐lung tissue interfaces in the treatment field may have a substantial effect on the calculated dose differences between the PB and MC algorithms. The PTV volume was 88 cc in our study, and the calculated differences between the PB and MC algorithms were 2% for V20, 3% for V10, and 3% for V5. Our results support the use of the MC algorithm for small treatment volumes.

## 5 CONCLUSION

In planning 3DCRT treatment, a homogeneous distribution of the mean dose in the target volumes of the GTV, CTV, PTV, and achieving high minimum doses is desirable. TPS, which is the only tool to create a radiation treatment plan, requires an accurate algorithm to accurately calculate the absorbed dose to assess the treatment plan before delivery. In this study, the absolute dose calculations by TLDs and the differences between the calculated absolute doses created by the PB and MC algorithms were evaluated by assessing the relevant DVH. In conclusion, the results are generally in good agreement with the TLD measurements. However, differences between the doses were more evident in areas of inhomogeneous transitions close to and within critical organs. While both algorithms provided compatible results in calculating the target volume in 3DCRT, the MC algorithm was superior to the PB algorithm in a heterogeneous medium or in critical organ dose calculations.

Comparisons of the dosage data acquired in this study reveal that the MC algorithm calculations are closer to the prescribed 60 Gy dose for PTV, while the difference between the PB and MC algorithms was non‐significant. The major difference due to dose calculation techniques by TPSs was observed in the MLD with significant medium heterogeneity, leading us to recommend using the MC algorithm in such heterogeneous sites.

## CONFLICT OF INTEREST

The authors have no relevant conflicts of interest to disclose.
